# Physiologically Based Pharmacokinetic Modeling of Persistent Organic Pollutants for Lifetime Exposure Assessment: A New Tool in Breast Cancer Epidemiologic Studies

**DOI:** 10.1289/ehp.10917

**Published:** 2008-03-19

**Authors:** Marc-André Verner, Michel Charbonneau, Lizbeth López-Carrillo, Sami Haddad

**Affiliations:** 1 Département des sciences biologiques, Université du Québec à Montréal, Montréal, Québec, Canada; 2 INRS-Institut Armand-Frappier, Université du Québec, Laval, Québec, Canada; 3 Instituto Nacional de Salud Publica, Cuernavaca, Mexico

**Keywords:** breast cancer, epidemiology, exposure assessment, persistent organic pollutants, physiologically based pharmacokinetic modeling

## Abstract

**Background:**

Despite experimental evidence, most epidemiologic studies to date have not supported an association between exposure to persistent organic pollutants (POP) and breast cancer incidence in humans. This may be attributable to difficulties in estimating blood/tissue POP concentration at critical time periods of carcinogenesis.

**Objectives:**

In this work we aimed to develop a tool to estimate lifetime POP blood/tissue exposure and levels during any hypothesized time window of susceptibility in breast cancer development.

**Methods:**

We developed a physiologically based pharmacokinetic (PBPK) model that can account for any given physiologic lifetime history. Using data on pregnancies, height, weight, and age, the model estimates the values of physiologic parameters (e.g., organ volume, composition, and blood flow) throughout a woman’s entire life. We assessed the lifetime toxicokinetic profile (LTP) for various exposure scenarios and physiologic factors (i.e., breast-feeding, growth, pregnancy, lactation, and weight changes).

**Results:**

Simulations for three POPs [hexachlorobenzene, polychlorinated biphenyl (PCB)-153, PCB-180] using different lifetime physiologic profiles showed that the same blood concentration at 55 years of age can be reached despite totally different LTP. Aside from exposure levels, lactation periods and weight profile history were shown to be the factors that had the greatest impact on the LTP.

**Conclusions:**

This new lifetime PBPK model, which showed the limitations of using a single sample value obtained around the time of diagnosis for lifetime exposure assessment, will enable researchers conducting environmental epidemiology studies to reduce uncertainty linked to past POP exposure estimation and to consider exposure during time windows that are hypothesized to be mechanistically critical in carcinogenesis.

Exposure to ubiquitous persistent organic pollutants (POPs) such as polychlorinated biphenyls (PCBs), dichlorodiphenyldichloroethylene (DDE), and hexachlorobenzene (HCB) has attracted attention in breast cancer etiology. These compounds have high solubility in lipids and long half-lives in organisms, and are present in measurable amounts in human tissues, blood, and milk. Although the mechanistic actions of these chemicals in carcinogenesis remain unclear, studies showed that some POPs have the potential to promote cancer development in various experimental models such as rodents and human cell lines. *In vitro* assays using MCF-7 human breast cancer cells showed that POPs can promote cell proliferation (Andersson et al. 1999; Du et al. 2000; Soto et al. 1995). POPs have also been shown to inhibit epidermal growth factor withdrawal-induced apoptosis (Davis et al. 2001).

Despite the experimental evidence, controversy still exists regarding breast carcinogenic properties of POPs in humans. An epidemiologic study first suggested that DDE blood concentration may be an important etiologic factor in breast cancer (Wolff et al. 1993). This finding led epidemiologists to address further the issue of environmental exposure to POPs and their potential implication in breast cancer. In subsequent years, several environmental epidemiology studies on the subject were published and their findings were greatly variable. Reviews and meta-analyses concluded, on the lack of evidence to support the hypothesis, that POP exposure could be linked to an increase in female breast cancer risk (Calle et al. 2002; Laden et al. 2001; Lopez-Cervantes et al. 2004). On the other hand, some studies showed a positive correlation between POP levels and breast cancer incidence (Aronson et al. 2000; Charlier et al. 2003; Demers et al. 2002; Hoyer et al. 2000; Romieu et al. 2000).

The variability in conclusions among epidemiologic studies might arise from methodologic challenges. One conclusion relates to the lack of tools for past exposure to pollutants assessment (Brody and Rudel 2003). In most cases, biologic assessment of exposure has been limited to measurements of blood or tissue levels in samples collected around the date of diagnosis. It is uncertain that blood or tissue concentrations sampled a few years before diagnosis reflect the body burden during potentially critical time windows such as fetal, neonatal, and pubertal periods. Epidemiologic conclusions on the link between POP exposure and breast cancer development are still based on the premise that single sampling is indicative of past POP exposures, highlighting the need for tools to assess lifetime toxicokinetic profiles (LTPs).

Physiologically based pharmacokinetic (PBPK) modeling represents a possible approach to estimating POP exposure during specific time windows. PBPK models are mathematic representations of xenobiotic pharmacokinetics (i.e., processes of absorption, distribution, metabolism, and excretion) based on the physiologic and biochemical parameters of a given organism (e.g., humans) and the physicochemical properties of the selected xenobiotic (Krishnan and Andersen 2001). Such models allow the prediction of blood or tissue concentrations at a given time after a given dose of the xenobiotic. Various types of physiologic changes, which can be mathematically described within a PBPK model, may affect the kinetics of a compound in an individual throughout his or her life. Examples of relevant physiologic changes are body weight variations, excretion of POPs through lactation (Neville et al. 1991), and physiologic changes due to aging (Haddad et al. 2006; Price K et al. 2003; Price PS et al. 2003) or pregnancy (Gentry et al. 2002, 2003). PBPK modeling can also accommodate a variety of exposure scenarios such as changes in the lifestyle of the subject and geographic/temporal monitoring data on environmental levels of POPs. Thus, development of a PBPK model able to simulate exposure throughout life while considering such physiologic changes would be very useful to assess past exposure during critical time windows.

In the past, several PBPK modeling efforts have described the toxicokinetics of different POPs. Many of these models are based on the assumption that these chemicals, which are highly lipophilic, are distributed uniformly between blood and tissues according to their contents in lipids (for PCBs: Anderson et al. 1977; Emond et al. 2005; Lutz et al. 1977, 1984; Tuey and Matthews 1977, 1980a, 1980b; for dioxins: Carrier 1991, Maruyama et al. 2003; van der Molen et al. 1996; for HCB: Yesair et al. 1986). Other models have added diffusion limitation to the fat compartment [Belfiore et al. 2007 (mirex); Lee et al. 2002, 2007 (PCB-153); Parham and Portier 1998 (PCBs); You et al. 1999 (*p,p*′-DDE)] or diffusion limitation between erythrocytes and plasma [Lu et al. 2006 (HCB)] to their model structure to improve model predictions of animal kinetic data. Although the addition of diffusion limitation has definitely had an impact on the initial uptake phase on a short time scale, it is not likely to be an important determinant for the toxicokinetics on a scale spanning many years. Most agree that partitioning for these compounds is driven by lipid solubility, and this has been corroborated with human and rodent *in vivo* data (Haddad et al. 2000).

Our overall goal in this work was to develop an exposure assessment tool that could be used in breast cancer epidemiologic studies to estimate lifetime POP blood/tissue exposure and levels during any hypothesized time window of susceptibility in breast cancer development. The specific objectives were to build a generic PBPK model framework to simulate POP toxicokinetics for any given physiologic profile and exposure data, and to evaluate through model simulation the impact of exposure scenarios and different physiologic factors such as pregnancy, lactation, and body weight on the lifetime internal exposure profile and the blood POP concentration at 55 years of age, a surrogate time representing the age of diagnosis. For the purposes of this study, three POPs with half-lives varying from 6 to 27.5 years were chosen to run the simulations: HCB; 2,2′,3,4,4′,5,5′-heptachlorobiphenyl (PCB-180); and 2,2′,4,4′,5,5′-hexachlorobiphenyl (PCB-153).

## Methods

The development of this new PBPK model framework for lifetime POP exposure in women can be separated into three distinct phases: model representation, model parameterization, and simulations. Model validation could not be achieved because of the evident lack of data—namely, a lifetime follow-up study measuring blood concentration at different moments and controlling all the input parameters. The model was coded using Advanced Continuous Simulation Language (ACSLXtreme, Aegis Technologies Group, Inc., Huntsville, AL, USA) and is available on request.

### Model representation

**Figure 1:**
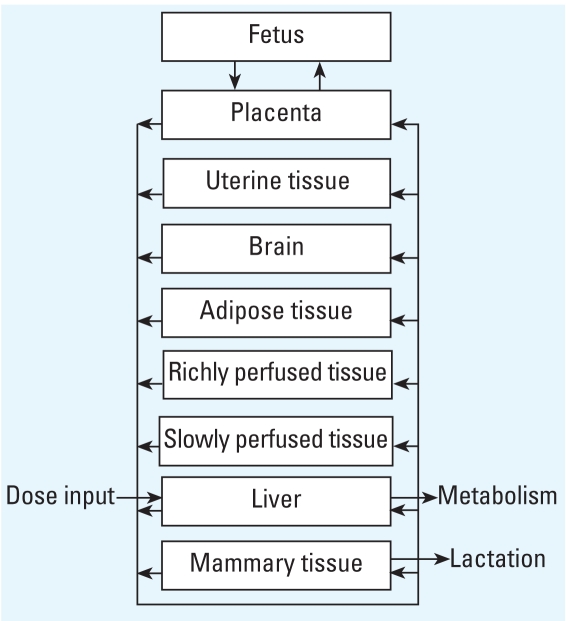
Conceptual representation of the PBPK model.

For this model, the woman is best functionally described as a network of nine tissue compartments perfused by blood circulation (Figure 1). Compartments are chosen for their known relevance to POP kinetics or in light of objectives of this study. The liver is set as the compartment where both intake via ingestion and metabolism (i.e., first-pass organ) take place. Because POPs are highly hydrophobic compounds, adipose tissue is defined as a compartment where significant chemical storage can occur. Because mammary tissue is the cancer site studied and is also an excretory organ (i.e., via lactation), it is also described as a separate compartment. Along with mammary and adipose tissues, uterine tissue, placenta, and fetus are represented as compartments because of their variations during pregnancy and postpartum periods (i.e., mainly volume change). The brain is added as a potentially interesting organ for the study of cognitive and motor effects of POPs. Finally, remaining tissues or organs, except for teguments (i.e., bones, nails, hair, cartilage), are grouped into slowly perfused tissues (mainly the skin, skeletal muscles, and heart) and richly perfused tissues based on their volume:blood flow ratio.

#### Absorption

Absorption of POPs can occur through different routes, but primarily occurs through food intake. For the purpose of this study, total intake is limited to a direct input into the liver compartment, and each POP is assumed to be completely bioavailable through the gastrointestinal tract, thus involving a hepatic first pass.

#### Distribution

The distribution of POPs is managed by blood flow to different compartments and partitioning from blood to tissues. This process is described by using mass balance differential equations (MBDEs) that assume homogenous distributions in tissues, as follows:


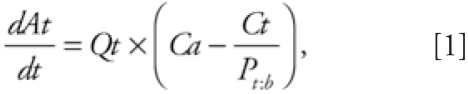


where *At* represents the amount of chemical in the compartment, *Qt* is the blood flow perfusing the compartment, *P* *_t:b_* is the tissue: blood coefficient for the compartment, and *Ca* and *Ct* are concentrations in arterial blood and the tissue, respectively.

#### Metabolism

Metabolism is assumed to be essentially limited to the liver compartment, and the rate is described by the product of the hepatic extraction ratio (*Eh*), the liver blood flow (*Ql* ), and the arterial blood concentration (*Ca*) entering the compartment, as follows:


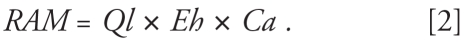


The value of *Eh* can be calculated from available data such as half-life values, Michealis-Menten constants (Vmax and Km), or intrinsic clearances. The MBDE in the liver therefore becomes


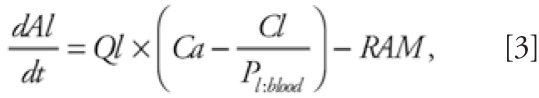


where *Al*, *Cl*, and *P* *_l:blood_* are the amount, concentration, and tissue:blood partition coefficient of the POPs in the liver, respectively.

#### Excretion

Because most POPs are poorly metabolized, the main elimination route occurs through excretion of unchanged chemicals. The model is adapted for two excretion pathways: lactation and parturition. POP excretion via lactation is represented as an output from the mammary tissue compartment through a partitioning process between mammary tissue and milk, and milk withdrawal by the suckling such as described by Lee et al. (2007) for PCBs in rats. This partitioning process is further addressed in the model parameterization section. The POP excretion via lactation is described as follows:


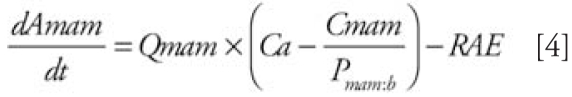


and


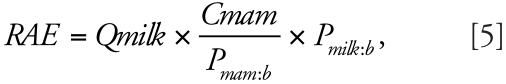


where *Amam* and *Cmam* are the POP amount and concentration in mammary tissue. *Qmam* is the blood flow to mammary tissue compartment, *Qmilk* refers to the milk flow out of the breast (i.e., the volume drunk per hour by infant in liters per hour), and *P* *_mam:b_* and *P* *_milk:b_* are the mammary:blood and milk:blood partition coefficients.

The placental transfer to the fetus is described using published equations in Gentry et al. (2002). The elimination of chemicals through parturition is described as a punctual extraction of the baby body and placenta burdens at the time of birth.

### Model parameterization

To simulate internal exposure in women throughout their entire life, compartments size, blood flows, and biochemical properties are described as variables that change as a function of age, body weight, body height, and pregnancy periods (see Supplemental Material online at http://www.ehponline.org/members/2008/10917/suppl.pdf). Mathematical equations describing these variable parameters are arranged so that information on body weight and body height in relation to age, collected from questionnaires, can be easily used as inputs throughout the entire simulation. Volume and blood flow parameters for liver, richly perfused, slowly perfused, and adipose tissue compartments are taken from Haddad et al. (2006). The equations describing early stages of development are used to calculate organs growth for the 0–1 year interval, because no data are available for that period. Because of the lack of data, uterine tissue and mammary tissue are set as a function of body weight and age (Gentry et al. 2002). Mammary tissue volume is assumed to start from 0 L at 10 years of age and increase linearly to its final volume at 14 years of age.

Physiologic changes during pregnancy are considered for the uterine tissue, mammary tissue, adipose tissue, placenta, and fetus compartments based on time elapsed since the beginning of pregnancy, as described by Gentry et al. (2002). Postpartum changes are set as a 6-month linear return to normal in the volume of organs influenced by pregnancy (Gentry et al. 2003). Blood flow to these compartments varies proportionally to their volume throughout pregnancy and postpartum changes. Lactation parameters are also allowed to change over the lactation period. Equations describing changes in daily excreted milk volume and milk lipid content as a function of postnatal time are taken from Neville et al. (1991) (see Supplemental Material online at http://www.ehponline.org/members/2008/10917/suppl.pdf). Placental diffusion constant (PAF) describing the exchange between the mother and the baby is given an arbitrary value of 1, because the model outputs in the woman (i.e., blood concentrations) are virtually not influenced by this value.

Tissue:blood and milk:blood partition coefficients are estimated using Poulin and Krishnan’s (1996) approach based on tissue water and lipid composition (Price K et al. 2003):


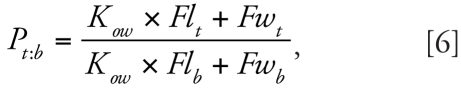


where *K* *_ow_* represents n-octanol:water partition coefficient, and *Fl* and *Fw* stand for the lipid and water fraction, respectively, for either tissue (subscript t) or blood (subscript b). Tissue composition is taken from Price K et al. (2003) (see Supplemental Material online at http://www.ehponline.org/members/2008/10917/suppl.pdf). Slowly perfused tissues compartment composition is calculated as scaled lipid and water content of muscles, skin, and heart taken from the same paper as follows:


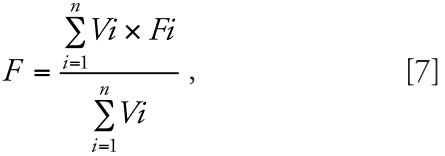


where *F* is the fraction of either lipid or water for the whole compartment, whereas *Fi* and *Vi* are the fraction of either lipid or water and the volume for organs included in the compartment, respectively. Richly perfused tissues compartment composition is calculated with the same equation (Equation 7). The richly perfused tissue compartment includes the lungs, kidneys, reproductive organs, spleen, glands, intestinal tract, and stomach tissues. The organ volumes (*Vi*) used for the calculation of this compartment composition are those at 18 years of age calculated with the equations in Haddad et al. (2001). The partition coefficients for richly and poorly perfused tissues compartments are calculated with Equation 6 using these scaled composition parameters. Because of the lack of data, the richly perfused tissues compartment partition coefficient is used for the mammary tissue, placenta, and uterine tissue compartments. Because no information is available on fetus compartment composition, we assessed the impact of different partition coefficients on the model outputs. The lack of significant impact of the tissue:blood partitioning for the fetus compartment on the toxicokinetic profile of the mother led us to arbitrarily give it the partition coefficient of the richly perfused tissues compartment.

Metabolism is parameterized from half-life values for the compounds to be studied. This assumes that POP elimination is essentially attributed to hepatic clearance. First, the intrinsic clearance values per kilogram of liver (*CLint* *_C_*) are calculated for the physiologic parameters at the age of half-life [*HL*_(_ *_h_*_)_] measurements:


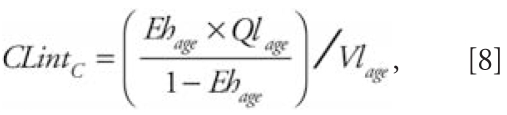


where


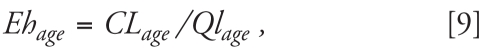



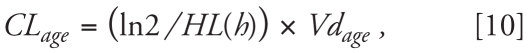



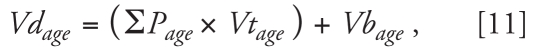


where *Ql* is the blood flow to the liver, *Vl* is the volume of the liver, *CL* is the clearance for the studied compound, *Vd* is the volume of distribution, *P* is the tissue:blood partition coefficient, *Vt* is the volume of tissues, and *Vb* is the volume of blood. The subscript *age* means that the parameters are calculated with the physiologic features at the age of individuals sampled for half-life value measurements, whereas the subscript (*h*) means that the values of half-lives were in hours. Extraction ratios are calculated from *CLint* *_C_*, which is assumed to be age invariant, and from the liver volume and blood flow, which change as a function of age, as follows:


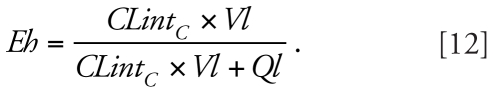


The liver weight–adjusted hepatic extraction ratio is used for the calculation of the metabolism rate. Although recent modeling studies (e.g., Clewell et al. 2004) introduced gene ontology data in their model to reflect age variations in intrinsic clearance, this was not considered in the present simulations because of the relative little impact it has shown on lifetime kinetics and on the data interpretation in this study (simulations not shown). If needed in the future, such information can easily be incorporated into the model.

### Model simulations

**Figure 2:**
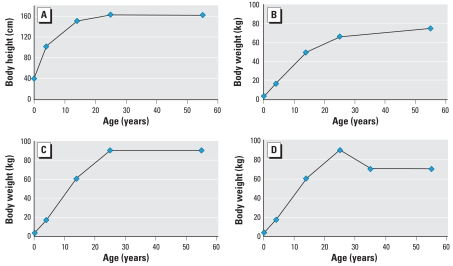
Body weight and body height profiles used for the simulations: (*A*) body height, (*B*) normal weight, (*C*) overweight, and (*D*) weight loss profiles. The normal weight and overweight scenarios represent linear increases in weight from 50 kg at 14 years of age to 70 and 90 kg, respectively, at 25 years of age. Weight loss scenario followed the overweight profile with a drop from 90 to 70 kg on a 10-year interval between the ages of 25 and 35 years.

Different scenarios were simulated to assess the impact of different physiologic processes or changes that can occur during the lifetime of a woman on the toxicokinetic profile of POPs as well as on the blood concentration at age of diagnosis. The input parameters that were changed for the simulations were breast-feeding in childhood, level of exposure through food intake, body weight and body height in function of age, number of pregnancies, age at child birth(s), lactation periods, and chemical properties for the chosen pollutants (log *K*_ow_ and hepatic extraction ratio). All simulations used as input the normal body weight profile and the height profile depicted in Figure 2, unless stated otherwise.

Three pollutants were chosen for the present studies: PCB-180, PCB-153, and HCB, chosen for their relevant concentrations found in human blood and adipose tissue as well as the fact that they differ in their *K*_ow_ and half-life. Log *K*_ow_ was 6.72 for PCB-153, 7.21 for PCB-180, and 5.73 for HCB [Agency for Toxic Substances and Disease Registry (ATSDR) 2000, 2002]. Hepatic extraction ratios were calculated as described in the parameterization section from the approximate half-lives of the chemicals, which were 27.5 years for PCB-153, 9.9 years for PCB-180, and 6 years for HCB in humans (ATSDR 2000; To-Figueras et al. 2000).

Although the actual food consumption levels of these POPs declined from the 1970 to the 1990s, it was kept constant for the purpose of the modeling effort. However, the actual levels may be entered as a variable into the model. For all simulation scenarios, the level of exposure through ingestion of contaminated food was set as a background daily exposure of 10 ng/kg body weight/day (unless specified otherwise).

#### Impact of breast-feeding in childhood

In this first set of simulations, the period of breast-feeding was set to 6 months, with a constant concentration of 2 μg/L to compare the kinetics of the three chemicals. The volume of milk ingested was modeled with the same equation used for the breast-feeding periods of the exposed woman. The milk concentrations used in these simulations were arbitrarily chosen and do not necessarily reflect specific actual levels of contaminants, although similar POP milk levels were found in the literature (Dewailly et al. 1996; Solomon and Weiss 2002). The purpose is simply to show how breast-feeding in childhood will impact the LTP of a given individual.

#### Impact of body weight change

For some simulations, the body weight parameter was varied to investigate the influence of adipose tissue volume and its variation throughout the lifetime of a woman on the tissue pollutant concentration. Body weight and body height profiles used in this study are depicted in Figure 2. The normal weight and overweight scenarios represented linear increases in weight from 50 kg at 14 years of age to 70 and 90 kg, respectively, at 25 years of age. Weight loss scenario followed the overweight profile with a drop from 90 to 70 kg on a 10-year interval between 25 and 35 years of age.

#### Impact of pregnancy and lactation

We performed simulations for several pregnancy history scenarios. The number of pregnancies was either one or two. Lactation period length effect on tissue or blood concentration was also assessed; lactation periods chosen for these simulations were 6 and 12 months.

#### Differences in toxicokinetic profiles for a given blood concentration at the age of diagnosis

For a given POP blood concentration, toxicokinetics profiles were obtained for simulations in women having different physiologic histories (i.e., different number of pregnancies, period of lactation, weight profile, and exposure). This was done to assess how much the lifetime internal exposure can differ for a given POP blood concentration at the age of diagnosis. The exposure values were optimized to reach the same blood concentration at 55 years of age for different physiologic histories.

## Results

### Impact of breast-feeding in childhood

**Figure 3:**
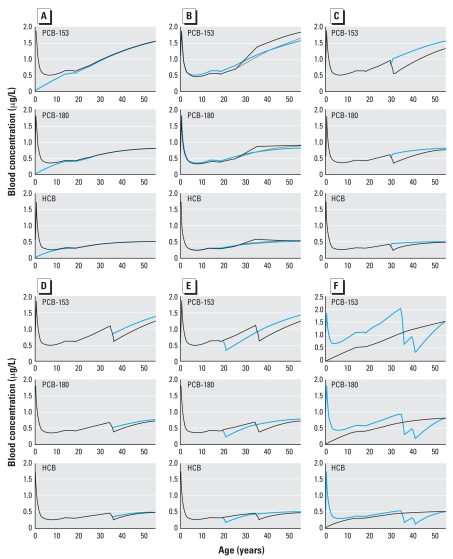
Toxicokinetic profiles for PCB-153, PCB-180, and HCB blood concentration for (*A*) normal body weight history and 10 ng/kg/day exposure to each of these chemicals for a woman who was not breast-fed in childhood (blue line) or breast-fed for 6 months (black line); (*B*) normal weight (gray line), weight loss (black line), or overweight (blue line) profiles and 10 ng/kg/day exposure to each of these chemicals for women who were breast-fed for 6 months in childhood; (*C*) normal body weight history and 10 ng/kg/day exposure to each of these chemicals for a woman who was breast-fed for 6 months in childhood and had a pregnancy at 30 years of age followed by no lactation (blue line) or a 12-month lactation period (black line); (*D*) normal body weight history and 10 ng/kg/day exposure to each of these chemicals for a woman who had a pregnancy at 35 years of age followed by a 6-month lactation period (blue line) or a 12-month lactation period (black line); (*E*) normal body weight history and 10 ng/kg/day exposure to each of these chemicals for a woman who was breast-fed for 6 months in childhood and had a pregnancy followed by a 12-month lactation period at 20 years of age (blue line) or 35 years of age (black line); (*F*) normal body weight history for a woman who was exposed to 10 ng/kg/day of each of the three chemicals and had no pregnancy (black line) or was breast-fed for 6 months in childhood, was exposed to 18.7 ng/kg/day PCB-153, 13.8 ng/kg/day PCB-180, 11.6 ng/kg/day HCB, and who had two pregnancies at 35 and 40 years of age followed by 12-month lactation periods (blue line).

We used the PBPK model to investigate the potential impact of additional early exposure through breast-feeding in childhood. For this purpose, a scenario considering breast-milk drinking for a period of 6 months was compared with a scenario where the woman was not breast-fed (Figure 3A). The milk concentration was set at 2 μg/L, and the background oral exposure was set as a daily intake of 10 ng/kg body weight. Scenarios were simulated for a woman with a normal body weight profile. For the breast-fed female scenario, the blood concentration at the end of the milk-drinking period reaches 1.87 μg/L for PCB-153, 1.81 μg/L for PCB-180, and 1.74 μg/L for HCB. These concentrations are even higher then those at the age of diagnosis (i.e., 55 years of age). At 5 years of age, the difference between the two scenarios was important for all three pollutants, because the blood concentration was about 2-fold higher for the breast-fed woman. However, by 20 years of age, these differences almost completely disappeared. It can also be observed that blood concentrations at 55 years of age cannot distinguish breast-fed from non-breast-fed individuals, even though internal concentrations in the breast-fed group were very high in infancy.

### Impact of body weight change

We also assessed the impact of body weight on POP kinetics with the use of various profiles shown in Figure 2. All these simulations used the same body height profile (Figure 2). Using these three physiologic profiles, simulations considering a 6-month period of milk drinking in childhood (milk concentration, 2 μg/L) and a daily exposure to 10 ng/kg of the chemicals were performed for the three chemicals studied (Figure 3B). Simulations show that both the normal and the overweight scenarios display similar kinetic profiles, despite the fact that PCB blood concentration at 55 years of age is slightly higher in the overweight profile than for the normal weight profile. A weight loss between 25 and 35 years of age raises the immediate blood POP concentrations considerably. The effect is more pronounced and lasts up to 55 years of age for PCB-153, where the blood concentrations at 35 years of age are 1.13 μg/L and 1.38 μg/L for the normal weight and weight loss profiles, respectively.

### Impact of pregnancy and lactation

We also investigated the impact of pregnancy and subsequent breast-feeding on blood POP concentrations. First, scenarios with pregnancy alone and pregnancy followed by a 12-month lactation starting at 30 years of age were simulated to compare the respective effect of these two factors (Figure 3C). For PCB-153, the blood concentration at 31 years of age is much lower in the woman who lactated for 12 months (0.53 μg/L blood) than in the woman without lactation (1.01 μg/L blood). The difference is still present at 55 years of age, although smaller than at 31 years of age. For PCB-180, lactation has a small effect on the blood concentration at 55 years of age, whereas for HCB it has no impact. Simulations show that the pregnancy alone induces a small drop in the blood concentration that rapidly returned to prepregnancy levels after postpartum physiologic changes (Figure 3C, normal lines).

We assesssed the impact of the length of the lactation period. Simulations for two lactation period lengths show that longer lactations have a greater impact on the blood concentration (Figure 3D). For PCB-153, the blood concentration at 55 years of age is 1.39 μg/L for a 6-month lactation and 1.25 μg/L for a 12-month lactation starting at 35 years of age. The difference between the two lactation periods is small for PCB-180 and negligible for HCB when only blood concentration at 55 years of age is considered.

POP toxicokinetic profiles were also compared for lactations of the same duration but held at different ages. Two scenarios were compared: child birth at either 20 or 35 years of age, followed by a 12-month breast-feeding period (Figure 3E). As expected, POP kinetics differ between the two scenarios. The effect of a lactation period later in life influences significantly the blood concentration at 55 years of age for PCB-153, whereas this effect is minimal for PCB-180 and practically absent for HCB in the simulated scenarios.

### Differences in toxicokinetic profiles for a given blood concentration at diagnosis

To demonstrate that the same blood concentration at 55 years of age can be the result of completely different kinetic profiles, we compared two scenarios with different lifetime profiles (Figure 3F). The first scenario represents a woman who was not breast-fed in childhood, was never pregnant, and was exposed throughout life to the background level of 10 ng/kg/day for each of the three pollutants. The second scenario is that of a woman who was breast-fed for 6 months with breast milk at a POP concentration of 2 μg/L, had two pregnancies, one at 35 and one at 40 years of age, followed by a 12-month lactation period each time. To obtain a final concentration at 55 years of age identical to the one in the first scenario, the daily exposure levels were optimized to the following levels: 18.7 ng/kg/day for PCB-153, 13.8 ng/kg/day for PCB-180, and 11.6 ng/kg/day for HCB. These simulations yielded completely different kinetic profiles despite resulting in the identical blood concentration at 55 years of age (Figure 3F). The blood concentration at 34 years of age showed the greatest difference, especially for PCB-153, where approximately a 2-fold higher level was calculated for the breast-feeding/pregnancy/higher exposure scenario (2.03 μg/L blood) than for the no breast-feeding/no pregnancy/background exposure scenario (1.07 μg/L blood).

## Discussion

Over the last decades, many environmental epidemiology studies have focused on the possible link between exposure to POPs and the development of breast cancer, but no clear overall conclusion could be drawn from the different findings. Discrepancies among conclusions from the various studies might be related to the false assumption that a unique late-life sampling reflects lifetime POP exposure. Our study supports this contention and proposes a new tool that could reduce this uncertainty in exposure assessment by simulating lifetime toxicokinetics of POPs in women.

The PBPK model built in this study can overcome exposure assessment problems by simulating normal development (i.e., growth, blood flows) and various historical events within the lifetime of a woman [i.e., breast feeding, changes in body mass index (BMI), pregnancy] to obtain the lifetime toxicokinetics of POPs. Using this model, we simulated several exposure and physiologic scenarios to assess the impact of different parameters on POP blood concentrations from 0 to 55 years of age.

Although *in utero* exposure is known to occur through placental diffusion, body burden at birth was set to 0. This methodologic choice relies on two facts: *a*) fetus tissue concentration estimation would require information on the exposure of the mother to simulate the placental transfer, and *b*) the baby’s body burden at birth is rapidly diluted by increasing tissue volumes and therefore has a small impact on lifetime toxicokinetics when compared with breast milk consumption (Clewell et al. 2004; Kreuzer et al. 1997).

Simulations showed that although pregnancy alone did not have a strong impact on blood POP concentrations, lactation exerted major changes in the toxicokinetic profiles. The longer and later in life a lactation period occurs, the greater its impact on blood POP concentration of the woman at 55 years of age. Thus, quantitative information on lactation is critical when evaluating past exposure to POPs. Moreover, simulations showed that body weight variations throughout life seemed to have a greater impact on blood POP concentrations than body weight level itself. A loss of weight can be regarded as a decrease in the adipose tissue volume in which POPs are preferentially stored, a phenomenon that leads to the unloading of POPs into blood. It has been previously shown that PCB-153, HCB, β-hexa-chlorocyclohexane, *p,p*′-DDE, and Aroclor 1260 levels in blood increase with weight loss (Imbeault et al. 2002). Therefore, BMI changes should be regarded as an important factor in POP kinetics. New approaches in exposure assessment that consider physiologic parameters such as BMI and lactation were developed by Wolff et al. (2005) with the use of first-order pharmacokinetic and predictor-based multivariate models. They concluded that possible exposure misclassifications in epidemiologic studies can occur if the impacts of BMI and lactation on POP concentrations are ignored. In accordance with such results, the current study clearly showed that sampling at the age of diagnosis is a questionable end point for lifetime exposure estimation. A more recent study from Wolff et al. (2007) stresses the importance of considering pharmacokinetic variability in epidemiologic studies. PBPK modeling as proposed here is particularly well suited for such considerations The use of PBPK models such as the one reported in our manuscript go much further than Wolff’s strategy in considering pharmacokinetics. This new approach is being proposed to epidemiologists. Instead of simply proposing pharmacokinetic factors such as BMI as other covariables in the epidemiologic studies, we suggest directly using PBPK model estimates of blood or tissue concentrations during different periods of life for each subject of the study to analyze if there is a relationship between disease and internal exposure.

We investigated the impact of breast milk consumption in childhood on internal exposure by comparing POP venous concentration in breast-fed and a bottle-fed women. Results showed that early-life blood POP concentrations are strongly influenced by breast-milk drinking for the first years of life, but that these effects are almost fully attenuated by 20 years of age. These findings are supported by a study on a Faroese birth cohort in which the primary contributors to the serum total PCB concentrations at 7 and 14 years of age were breast-feeding and blubber consumption, respectively (Barr et al. 2006). The work reported herein showed that blood or tissue POP concentration at the age of diagnosis does not reflect the important body burden resulting from early life breast-feeding, a possibly important time window of exposure.

By simulating the lifetime blood concentrations for two distinct exposure and life history scenarios with the same level at 55 years of age, this study showed the poor predictive value of late-life sampling for past exposure assessment. Late lactations can dramatically decrease POP concentrations and lead to lower blood concentrations at the age of diagnosis, even for a high-exposure profile. To eliminate this artifact, it is crucial that the estimation takes into account such physiologic events.

This PBPK model enables the consideration of chemical-specific parameters affecting distribution (log *K*_ow_) and elimination (half-life). The simulations performed with the three contaminants showed that blood PCB-153 levels were more sensitive to the main factors—that is, lactation and body weight change—than those for PCB-180 or HCB. This can be explained by their different half-lives, a parameter that strongly correlates with the time required for the pollutant to reach steady state in the body. Chemicals with a shorter half-life reach steady state more rapidly, leading to the faster attenuation of the impact that these physiologic events may have on blood concentration. On the other hand, the log *K*_ow_ is unlikely to account for differences in POP toxicokinetics, because it has been reported that compounds with a log *K*_ow_ value > 4 will partition similarly between blood and organs (Haddad et al. 2000). Like physiologic parameters, interindividual variability also exists in half-life values as well as blood and adipose tissue lipid content, which are sensitive parameters of the PBPK model (see Supplemental Material online at http://www.ehponline.org/members/2008/10917/suppl.pdf). Apart from doing a toxicokinetic study in each individual, there currently exists no method to estimate half-lives in individuals. Similarly, determining lipid fractions in blood and adipose tissue of individuals represents another difficulty, and such a practice can be very costly. Using average values of these parameters is an acceptable surrogate, because they lead to prediction errors under a 1.5-fold difference in blood concentrations in the case of intrinsic clearance and 1.8-fold in the case of lipid composition in adipose tissues and blood (see Supplemental Material online at http://www.ehponline.org/members/2008/10917/suppl.pdf).

The PBPK model developed herein refines the assessment of past tissue exposure by incorporating physiologic processes that greatly affect POP kinetics. The use of such a tool should permit epidemiologists to better assess past exposure and to investigate the potential critical windows of exposure to POPs in cancer development, a commonly reported concern in epidemiology studies. The importance of exposure assessment for different critical time windows is supported by studies on breast cancer incidence among Japanese women who were exposed to radiation; these studies show that exposure at a lower age has a higher impact on cancer development than exposure at later-life stages (Hoel and Dinse 1990; Tokunaga et al. 1994). Moreover, a recent study reported that exposure to *p,p*′-DDT early in life may increase the risk of breast cancer (Cohn et al. 2007). By using estimated LTPs and internal exposure levels for different time frames, this hypothesis could be further addressed.

Furthermore, the toxicity of certain POPs may not be attributable entirely to parent compound. Some evidence indicates that metabolites may also elicit a biochemical or toxic response (Machala et al. 2004; Meerts et al. 2004; Safe 1994; You et al. 2006). Because the PBPK model describes the metabolism of the chemicals, the amount of metabolites formed in different periods of life can be assessed and used for epidemiologic analysis in the same way that we use internal concentrations of the parent compound. Thus, PBPK modeling can also add another dimension to POP epidemiologic studies.

To perform simulations, information on the proposed important variables to be used as inputs in the model must be gathered within the epidemiologic questionnaire. The model was constructed so that it requires information on body weight and height as a function of age. Information on pregnancy and lactation periods is also a prerequisite to model simulation. This information must be associated with the age of the woman at birth of her children and the duration of the lactation periods. The model can easily include any information on exposure levels that could vary as a function of changes in dietary lifestyle (e.g., increase in fish consumption or in fatty foods for certain periods of life) as well as geographic/temporal monitoring data on environmental levels of selected pollutants. Once these parameters are included in the model, the exposure scenario can be optimized with the use of information on both estimated exposure and blood or tissue samples.

Although the proposed PBPK model framework for POP lifetime exposure assessment in women is constructed on validated descriptions of absorption, distribution, metabolism, and excretion of various POPs in rodents or humans, further studies are needed to validate the model. The model proposed herein can be used as a generic framework in which adjustments/modifications can be made to incorporate additional toxicokinetic processes that may be specific to particular POPs (e.g., diffusion limitation, plasma or tissue protein binding) if relevant for a lifetime scale in humans.

## Conclusion

This study is the first to propose a PBPK modeling approach for the assessment of lifetime internal exposure to POPs in the context of epidemiologic studies. The proposed model has the potential to be used in environmental epidemiology research to reduce the uncertainty in past tissue exposure estimation. This approach can not only strengthen the validity and reproducibility of studies on the impact of POPs on breast cancer incidence in humans, but also help to assess the effect of exposure during critical time windows in breast cancer development and other late-life diagnosis pathologic end points.
