# Erratum: “Physiologically Based Pharmacokinetic Modeling of Persistent Organic Pollutants for Lifetime Exposure Assessment: A New Tool in Breast Cancer Epidemiologic Studies”

**DOI:** 10.1289/ehp.121-a181

**Published:** 2013-06-01

Verner et al. have reported an error in their article “Physiologically Based Pharmacokinetic Modeling of Persistent Organic Pollutants for Lifetime Exposure Assessment: A New Tool in Breast Cancer Epidemiologic Studies” [Environ Health Perspect 116:886–892 (2008)]. In the legend for Figure 3, the descriptions for *D* and *E* were inverted. The corrected figure legend is as follows:

Figure 3 - Toxicokinetic profiles for PCB-153, PCB-180, and HCB blood concentration for (*A*) normal body weight history and 10 ng/kg/day exposure to each of these chemicals for a woman who was not breast-fed in childhood (blue line) or breast-fed for 6 months (black line); (*B*) normal weight (gray line), weight loss (black line), or overweight (blue line) profiles and 10 ng/kg/day exposure to each of these chemicals for women who were breast-fed for 6 months in childhood; (*C*) normal body weight history and 10 ng/kg/day exposure to each of these chemicals for a woman who was breast-fed for 6 months in childhood and had a pregnancy at 30 years of age followed by no lactation (blue line) or a 12-month lactation period (black line); (*D*) normal body weight history and 10 ng/kg/day exposure to each of these chemicals for a woman who had a pregnancy at 35 years of age followed by a 6-month lactation period (blue line) or a 12-month lactation period (black line); (*E*) normal body weight history and 10 ng/kg/day exposure to each of these chemicals for a woman who was breast-fed for 6 months in childhood and had a pregnancy followed by a 12-month lactation period at 20 years of age (blue line) or 35 years of age (black line); (*F*) normal body weight history for a woman who was exposed to 10 ng/kg/day of each of the three chemicals and had no pregnancy (black line) or was breast-fed for 6 months in childhood, was exposed to 18.7 ng/kg/day PCB-153, 13.8 ng/kg/day PCB-180, 11.6 ng/kg/day HCB, and who had two pregnancies at 35 and 40 years of age followed by 12-month lactation periods (blue line).

The authors regret the error.

